# 2427. Characterization of Early Healthcare-Associated Infections (HAI) After Implantation of Left Ventricular Assist Devices (LVAD)

**DOI:** 10.1093/ofid/ofad500.2046

**Published:** 2023-11-27

**Authors:** Kendall Kling, William J Moore, Teresa Zembower, Alison Jirak, Gretchen Nonog, Grace Magliola, Rebecca Harap, Jane Wilcox, Duc Pham, Tingqing Wu, Heather Byrd, Valentina Stosor

**Affiliations:** Northwestern University, Chicago, Illinois; Northwestern Medicine, Chicago, Illinois; Northwestern University, Chicago, Illinois; Northwestern University, Chicago, Illinois; Northwestern University, Chicago, Illinois; Northwestern University, Chicago, Illinois; Northwestern University, Chicago, Illinois; Northwestern University, Chicago, Illinois; Northwestern University, Chicago, Illinois; Northwestern University, Chicago, Illinois; Northwestern University, Chicago, Illinois; Northwestern University Feinberg School of Medicine, Chicago, Illinois

## Abstract

**Background:**

LVADs are established for therapy for heart failure and are implanted in > 2,500 patients in the US annually, but are complicated by infection in up to 60% of patients. Our aim was to characterize the spectrum and epidemiology of post-LVAD infections.

**Methods:**

We performed a single-center prospective observational study of 17 patients post-LVAD implantation from 7/2022 to 3/2023. Demographics, clinical, and microbiological data were collected as part of a study examining these changes in the cutaneous microbiome of the LVAD driveline (DL) site over the duration of LVAD support. Summary and descriptive statistics were performed.

**Results:**

Of the 17 patients, the mean age was 56.7, the majority were male, non-Hispanic White, and had chronic kidney disease (Table 1). Mean follow up was 136 days (range 17-265). VAD-related infections included 3 blood stream infections (mean post-LVAD date 42.3) (Table 2). There was a total of 8 episodes of hospital acquired pneumonia (HAP) in 4 unique participants. Urinary tract infections occurred in 35.3% (n=6), intra-abdominal infection in 17.6% (n=3), and skin and soft tissue infection in 5.9% (n=1). Multi-drug resistant organisms (MDRO) were detected in 8 participants, including vancomycin-resistant Enterococcus (n=1 for UTI and n=8 for rectal colonization), carbapenem-resistant Enterobacteriaceae (n=1), and *C. difficile* (n=3). Two participants expired due to heart failure (n=2) and infection (n=1).

**Figure:**
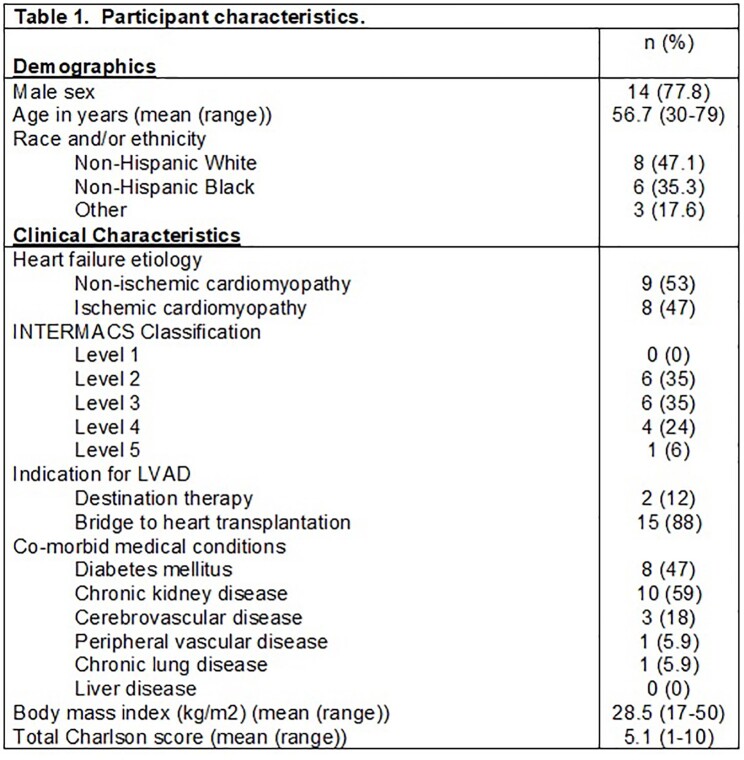

**Figure:**
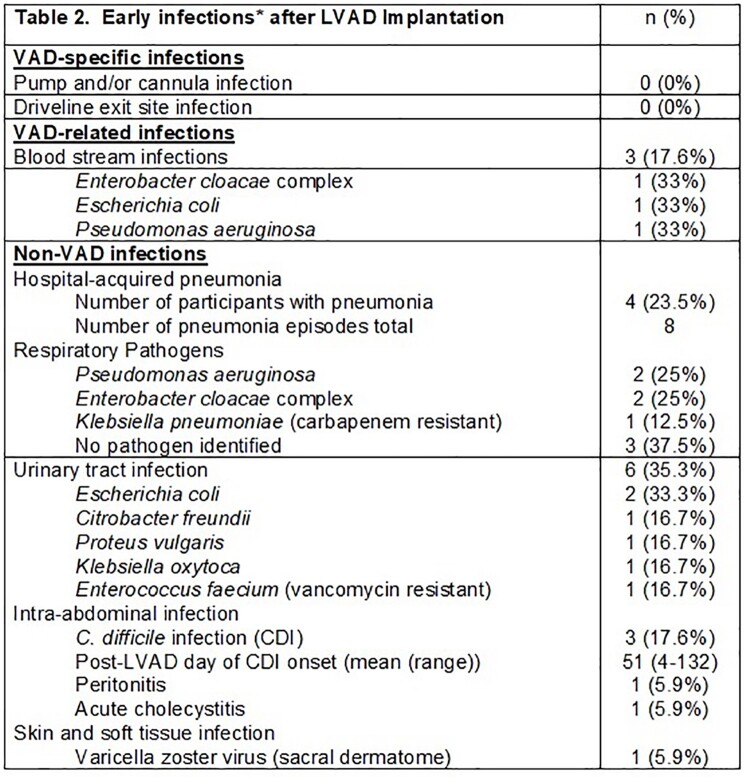

**Conclusion:**

We observed a high rate of early HAI post-LVAD implantation, including MDRO infections, and a strikingly high rate of VRE acquisition (47%). *C. difficile* infection rates were also high at 18%. Further study is needed to determine risks for MDRO infection and whether early HAI impact the LVAD DL microbiome and development of LVAD infection.

**Disclosures:**

**Valentina Stosor, MD**, DiaSorin S.p.A.: Advisor/Consultant|Eli Lilly & Company: Grant/Research Support

